# Urban-rural differences in daily time-activity patterns, occupational activity and housing characteristics

**DOI:** 10.1186/s12940-015-0075-y

**Published:** 2015-11-13

**Authors:** Carlyn J. Matz, David M. Stieb, Orly Brion

**Affiliations:** Air Health Effects Assessment Division, Health Canada, 269 Laurier Ave W, PL 4903C, Ottawa, ON K1A 0K9 Canada; Population Studies Division, Health Canada, 445-757 West Hasting St., Federal Tower, Vancouver, BC V6C 1A1 Canada; Population Studies Division, Health Canada, 50 Colombine Driveway, Tunney’s Pasture, PL 0801A, Ottawa, ON K1A 0K9 Canada

**Keywords:** Time-activity, Urban–rural, Occupation, Housing characteristics, Environmental exposure

## Abstract

**Background:**

There is evidence that rural residents experience a health disadvantage compared to urban residents, associated with a greater prevalence of health risk factors and socioeconomic differences. We examined differences between urban and rural Canadians using data from the Canadian Human Activity Pattern Survey (CHAPS) 2.

**Methods:**

Data were collected from 1460 respondents in two rural areas (Haldimand-Norfolk, Ontario and Annapolis Valley-Kings County, Nova Scotia) and 3551 respondents in five urban areas (Vancouver, Edmonton, Toronto, Montreal, and Halifax) using a 24-h recall diary and supplementary questionnaires administered using computer-assisted telephone interviews. We evaluated differences in time-activity patterns, occupational activity, and housing characteristics between rural and urban populations using multivariable linear and logistic regression models adjusted for design as well as demographic and socioeconomic covariates. Taylor linearization method and design-adjusted Wald tests were used to test statistical significance.

**Results:**

After adjustment for demographic and socioeconomic covariates, rural children, adults and seniors spent on average 0.7 (*p* < 0.05), 1.2 (*p* < 0.001), and 0.9 (*p* < 0.001) more hours outdoors per day respectively than urban counterparts. 23.1 % (95 % CI: 19.0–27.2 %) of urban and 37.8 % (95 % CI: 31.2–44.4 %) of rural employed populations reported working outdoors and the distributions of job skill level and industry differed significantly (*p* < 0.001) between urban and rural residents. In particular, 11.4 % of rural residents vs. 4.9 % of urban residents were employed in unskilled jobs, and 11.5 % of rural residents vs. <0.5 % of urban residents were employ in primary industry. Rural residents were also more likely than urban residents to report spending time near gas or diesel powered equipment other than vehicles (16.9 % vs. 5.2 %, *p* < 0.001), more likely to report wood as a heating fuel (9.8 % vs. <0.1 %; *p* < 0.001 for difference in distribution of heating fuels), less likely to have an air conditioner (43.0 % vs. 57.2 %, *p* < 0.001), and more likely to smoke (29.1 % vs. 19.0 %, *p* < 0.001). Private wells were the main water source in rural areas (68.6 %) in contrast to public water systems (97.6 %) in urban areas (*p* < 0.001). Despite these differences, no differences in self-reported health status were observed between urban and rural residents.

**Conclusions:**

We identified a number of differences between urban and rural residents, which provide evidence pertinent to the urban–rural health disparity.

## Background

Compared to urban and suburban populations, there is consistent evidence of a health disadvantage associated with living in rural areas. Research in Canada has identified higher mortality rates, decreased life expectancy, greater incidence and prevalence of morbidity, and poorer self-reported health status in rural populations [1–6]. For example, life expectancy at birth is at least 2 yrs less for men in rural areas compared to urban areas and the risks of death from circulatory disease or respiratory disease are as much as 10 % higher in rural areas [1]. This health disparity may be the result of differences in health risk factors, including health behaviours and socioeconomic status (SES) [1–5]. Additionally, differences in activity patterns between urban and rural populations may potentially lead to differences in exposure and risk(s) related to environmental contaminants, further contributing to the health disparity.

The Canadian Human Activity Pattern Survey (CHAPS) 2 was conducted by Health Canada to provide information on daily time-activity patterns, potential exposures to environmental contaminants, occupational activities, and housing characteristics [7]. This survey was undertaken to provide information to support exposure and risk assessment activities related to environmental health. While many studies are limited to urban areas, the target population for CHAPS 2 included residents from five major urban centres and two rural regions, allowing for comparisons between these groups to identify potential differences in risks or vulnerability.

Initial findings from CHAPS 2 indicated that people living in rural areas spend more time outdoors [7], which combined with potentially greater exertion in physically demanding agricultural and other primary industry occupations, could result in an increased dose of ambient air pollution. Air pollution is associated with many adverse health effects, including cardiovascular and respiratory diseases [e.g. 8, 9]. The Global Burden of Disease project reported that ambient particulate matter (PM) pollution accounted for over 3 million premature deaths and 3 % of global disability-adjusted life years [10]. Ambient PM was ranked as the ninth greatest risk factor for the global burden of disease. Among the top 20 risk factors, the only environmental risk factors were household air pollution (ranked third) and ambient PM. Canadian studies of health impacts associated with ambient air pollution have largely focused on urban areas [e.g. 11–14]. In comparison, the health effects of environmental exposures are not well documented for rural residents despite the potentially greater exposures for this population. In particular, rural residents may face greater exposures to air pollution from woodsmoke, which has been estimated to account for up to 70 % of ambient particulate matter in some provinces [15].

In addition to differences in exposure to air pollution, other factors such as occupation may also contribute to the health disparity between urban and rural populations. Rural residents may be employed in more hazardous occupations, such as agriculture and logging and forestry [16, 17]. Factors such as housing characteristics and personal behaviours may also contribute to differences in exposure to other environmental contaminants and associated health risks.

In this study, we evaluated potential sources of urban–rural disparities in health status, using the data collected in CHAPS 2, including daily time-activity patterns, occupational activity, and housing characteristics that may influence exposure to environmental and occupational hazards.

## Methods

### CHAPS 2 survey data

Detailed survey methodology for CHAPS 2 has been published previously [7]. Briefly, a random digit dialling survey was conducted, in 2010–2011, using computer-assisted telephone interview (CATI) technology to collect time-activity data and questionnaire responses. The target population was Canadian residents of all ages with a telephone residing in one of five urban areas (Vancouver, Edmonton, Toronto, Montreal, and Halifax) and one of two rural regions (Haldimand-Norfolk, Ontario and Annapolis Valley-Kings County, Nova Scotia). These rural regions were chosen for the survey due to their similar size, distance to large urban centres, similar agricultural industry of fruit production, and tendency to experience elevated concentrations of air pollution in relation to regional smog episodes. Delineations of urban and rural areas were adopted from reference materials prepared for the 2006 Canadian Census [18]. An urban area has a minimum population of 1000 people and population density of at least 400 people per square km; and rural areas include all territory outside urban areas. For the rural regions, screening questions were used to ensure that the respondents resided in rural portions of the regions and not in the urban areas (e.g. towns). The sampling frame was divided into two sub-frames, as a means to meet the objective of over-sampling infants (i.e. respondents less than 1 year of age). The infant survey included only respondents less than 1 year of age, while the non-infant survey randomly selected a household member older than 1 year of age. For the non-infant survey, if the household contained only adults (i.e. ≥18 years), a respondent was selected using the “next birthday” method, which allows for random selection of a participant without asking intrusive questions on household composition. For households consisting of both children (i.e. 1 to 17 years) and adults, a child was randomly selected 70 % of the time from all the children in the home, and the remaining 30 % of the time an adult was randomly selected from all the adults in the home. Oversampling of infants and children was performed as these groups may be more vulnerable to environmental pollutants [19], and there were few observations for these groups in CHAPS 1 [20].

The CHAPS 2 survey instrument was based on the original CHAPS surveys [20] and consisted of three main components: questions regarding respondent characteristics and household composition; a 24-h recall diary; and, a supplemental questionnaire covering activities related to exposures to specific contaminants, dwelling characteristics, SES, and health status. The survey instrument is available in Supplementary Materials of the previous publication [7]. The 24-h recall diary was used to collect time-activity information as respondents described their activities starting at midnight of the previous day. The CATI diary tool captured time, location, and activity for each sequential activity in the 24 h period. Location and activity information were matched to codes that had been used in CHAPS 1. This study was approved by Health Canada’s Research Ethics Board.

Sampling was split between summer 2010 and winter 2011. 5011 respondents participated in the survey, which included 3551 urban and 1460 rural respondents. Efforts were taken to sample each day of the week with the same frequency. Response rates were 12 % and 3 % for the non-infant and infant surveys, respectively. Of note, the infant survey response and refusal rate estimates should be considered very conservative and interpreted with caution. Many of the refusals likely corresponded to households that would have been considered out-of-scope, as many households declined to participate in the survey prior to determination if the household included an infant. Given the low prevalence of infants in the general population, the majority of these unverified homes likely would not include an infant. Also, low response rates do not necessarily lead to non-response bias [21] and proper weighting of telephone surveys can provide accurate information despite low response rates [22]. More generally, it is important to note that response rates have been decreasing in telephone surveys for the past several decades, with steeper declines in the more recent past [22]. Decreasing response rates may introduce a bias if respondents have different characteristics than non-respondents for variable(s) of interest. A greater proportion of CHAPS 2 respondents (aged 25–64) had a university or higher level of educational attainment compared to the Canadian population of the same age, which was similar to CHAPS 1, which had a higher response rate [7]. Survey weights were calculated to account for oversampling of certain age groups, adjustments for non-response, and to allow for generalization of survey results to the entire target area population.

### Analysis

The main purpose of this analysis was to evaluate possible differences between urban and rural populations in daily time-activity patterns, occupational activity and housing characteristics that may influence exposure to environmental and occupational hazards. We hypothesized that rural populations spent more time outdoors, including working outdoors, were more likely to be employed in primary industry, and were less likely to have air conditioning at home.

To facilitate analysis of time-activity patterns, the location information provided in the recall diaries was classified into four major groupings: indoors at home, other indoor locations, outdoors and in vehicle. The study sample was categorized according to six age groups: infants (<1 yr), young children (1–4 yrs), children (5–11 yrs), adolescents (12–19 yrs), adults (20–59 yrs), and seniors (60+ yrs).

To evaluate household SES, total household income was compared to low income cut-offs (LICOs), corresponding to participants’ family and community sizes. LICOs are threshold values, developed by Statistics Canada, that indicate incomes below which a household will likely devote a larger share of its income to food, shelter, and clothing compared to an average family [23]. LICO values are adjusted for family size and community size. Along with SES, other covariates chosen a priori include level of education, gender, and age.

To evaluate occupational skill level, job descriptors were coded according to the National Occupational Classification for Statistics (NOC-S). This classification system is based on the kind of work performed and groups are based on skill level [24]. To evaluate occupational industry, the job descriptors were coded according to the North American Industrial Classification System (NAICS). This classification system groups industries according to similarity in production processes used to produce goods and services and identifies the associated economic activity [25].

CHAPS 2 has complex design, including stratification by geographic region and season of data collection, as well as clustering by households. The complex design was accounted for in all the data analyses. To make the estimates representative of the CHAPS2 target population, all analyses were weighted using sampling weights. Differences between rural and urban populations were evaluated by fitting linear regression models to continuous variables and logistic regression models to categorical variables, with rural–urban indicator as predictor. Age, gender, education, occupation, SES, and smoking status were forced into these models individually and in combination. Where dependent variables pertained to households rather than individuals, only household SES was included as a covariate. Depending on the model type (linear or logistic), the coefficient for the rural–urban indicator was interpreted as difference in means or log-odds, respectively, of the response variable, between rural and urban populations. The coefficients were tested for significance using design-adjusted Wald F- and Chi-square tests with significance level α = 0.05 (unless otherwise indicated). Taylor linearization method was used to estimate sampling errors. The estimated sampling variability was evaluated against Statistics Canada’s guidelines [26]: estimates with high sampling variability were interpreted with caution and estimates with very high variability were suppressed. The data analysis was performed with SAS enterprise Guide 4.2 (SAS Institute Inc., Cary, NC) using SURVEYMEANS, SURVEYREG, SURVEYLOGISTIC and SURVEYFREQ procedures.

## Results

### Time-activity patterns

**Fig. 1:**
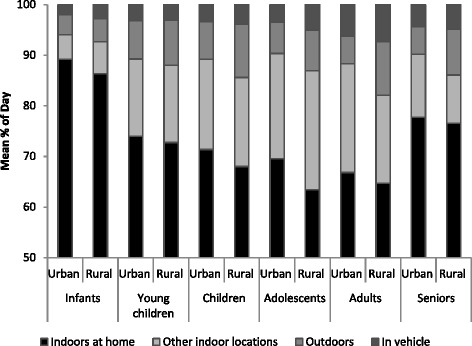
Time-activity patterns for urban and rural populations by age group. Bars represent population weighted estimates. Note: a minimum of 60 % of average daily time was spent indoors at home

For each age group, urban and rural populations reported spending a majority of daily time (>15 h/day) indoors at home (Fig. 1 and Table 1). Significant differences between the time that urban and rural residents spent outdoors were noted for children (5–11 yrs; *p* = 0.025), adults (20–59 yrs; *p* < 0.001), and seniors (60+ yrs; *p* < 0.001). Rural children, adults and seniors reported spending on average 0.7, 1.2, and 0.9 h more outdoors, respectively. The increase in daily time spent outdoors by the rural population corresponded to a decrease in daily time spent in other indoor locations for adults (*p* = 0.005) and seniors (*p* = 0.002), compared to those living in urban areas. Small but non-significant increases in time spent in vehicles were noted for rural vs. urban populations for each age group (except 1–4 yrs), despite potentially longer commute distances for school, work, and shopping activities.

**Table 1:** Daily time spent in different locations for the urban and rural population by age group

Age Group	Number	Community	Indoors at home	Other indoor locations	Outdoors	In vehicle
			Weighted average hours per day (95 % CI)			
Infants	116	Urban	21.4 (20.2–22.1)	1.2 (0.7–1.6)^d^	0.9 (0.4–1.5)^d^	0.5 (0.3–0.6)^d^
(<1 yr)	40	Rural	20.7 (19.6–21.8)	1.5 (0.8–2.3)^d^	1.1 (0.5–1.7)^d^	0.7 (0.4–0.9)^d^
Young Children	217	Urban	17.7 (17.0–18.5)	3.7 (3.0–4.4)	1.8 (1.2–2.4)^d^	0.8 (0.5–1.0)^d^
	52	Rural	17.4 (16.1–18.8)	3.7 (2.4–4.8)	2.1 (1.0–3.3)^d^	0.7 (0.5–1.0)^d^
(1–4 yrs)						
Children	316	Urban	17.1 (16.5–17.7)	4.3 (3.7–4.8)	1.8 (1.5–2.1)^a^	0.8 (0.6–1.0)
(5–11 yrs)	108	Rural	16.3 (15.7–17.0)	4.2 (3.5–4.9)	2.5 (1.9–3.1)	0.9 (0.7–1.1)
Adolescents	216	Urban	16.7 (15.7–17.6)	5.0 (4.1–5.9)	1.5 (1.0–2.0)^d^	0.8 (0.7–1.0)
(12–19 yrs)	114	Rural	15.2 (13.6–16.8)	5.6 (4.4–6.8)	1.9 (1.5–2.4)	1.2 (0.9–1.6)
Adults	1613	Urban	16.0 (15.6–16.4)	5.1 (4.7–5.5)^b^	1.3 (1.1–1.5)^c^	1.5 (1.3–1.7)
(20–59 yrs)	571	Rural	15.5 (14.9–16.2)	4.2 (3.6–4.7)	2.5 (2.1–3.0)	1.8 (1.4–2.1)
Seniors	1049	Urban	18.6 (18.2–19.0)	3.0 (2.7–3.3)^b^	1.3 (1.1–1.5)^c^	1.0 (0.9–1.2)
(60+ yrs)	568	Rural	18.4 (17.9–18.8)	2.3 (2.0–2.6)	2.2 (1.9–2.5)	1.2 (1.0–1.4)

Notes: Significant difference between urban and rural populations ^a^(*p* < 0.05), ^b^(*p* < 0.01), ^c^(*p* < 0.001)

^d^High sampling variability, interpret with caution

The increased time spent outdoors by adults and seniors living in rural areas may be attributable to time spent working outdoors instead of indoors. In the week prior to data collection, 23.1 % (95 % CI: 19.0–27.2 %) of urban and 37.8 % (95 % CI: 31.2–44.4 %) of rural employed populations reported working outdoors. Unadjusted odds of working outdoors were greater for rural vs. urban respondents (*p* < 0.001) and this difference was maintained with adjustment for education, gender, age and SES, alone or in combination. Unadjusted and adjusted odds ratios (OR) are provided in Table 2. Of those that worked outdoors in the previous week, the urban population average was 17.4 h (95 % CI: 14.8–20.1 h) and the rural population average was slightly greater at 22.4 h (95 % CI: 17.9–26.8 h); however, this difference was not statistically significant.

**Table 2:** Unadjusted and adjusted odds ratio (95 % confidence interval) estimates for the association between rural vs. urban residence and time-activity, chemical exposures, and housing characteristics (reference group is urban)

Adjustment	Respondent worked outdoors	Respondent exposed to engine exhaust (excluding vehicles)	Respondent exposed to solvents, fumes, or chemicals	Anyone in household smoked cigarettes^a^	Pesticide usage at household (indoor or outdoor)^a^	Household has air conditioning^a^
None	**2.0** ^b^	**3.7**	1.4	**1.8**	**1.6**	**0.6**
	(1.4–2.9)	(2.5–2.3)	(1.0–2.1)	(1.4–2.3)	(1.0–2.7)	(0.4–0.7)
Education	**1.9**	**3.7**	1.5	NA	NA	NA
	(1.3–2.9)	(2.4–5.9)	(1.0–2.3)			
Gender	**2.1**	**3.8**	1.4	NA	NA	NA
	(1.5–3.1)	(2.7–5.5)	(1.0–2.1)			
Age	**2.1**	**3.7**	1.4	NA	NA	NA
	(1.4–2.9)	(2.6–5.4)	(0.9–2.1)			
SES	**2.4**	**3.5**	1.3	**1.9**	**1.8**	**0.6**
	(1.6–3.5)	(2.3–5.3)	(0.8–2.0)	(1.5–2.5)	(1.0–3.1)	(0.4–0.8)
All	**2.2**	**3.8**	1.3	NA	NA	NA
	(1.5–3.3)	(2.5–5.7)	(0.8–2.1)			

^a^Data collected at household level, therefore only household level adjustments applied. ^b^Bold indicates statistical significance (*p* < 0.05)

### Occupation and income

**Fig. 2:**
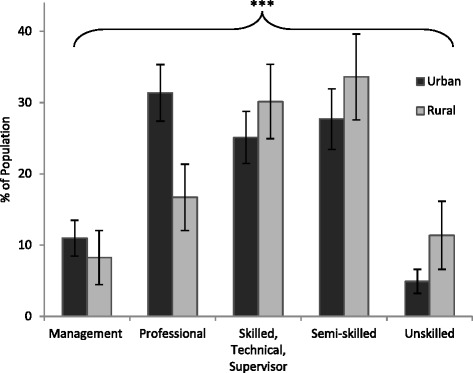
Occupational skill level, based on NOC-S classification, for urban and rural populations (≥18 yrs). Bars represent population weighted estimates ± 95 % CI. Difference between urban and rural is significant (***p < 0.001)

Based on the NOC-S classification system, occupational skill level for employed urban and rural respondents aged ≥18 yrs was compared (Fig. 2). In the urban population, over 30 % were classified as professional, compared to less than 20 % of the rural population. In both urban and rural populations, over 25 % were classified as skilled/technical/supervisor and semi-skilled categories, with slightly higher percentages in the rural population. Over 10 % of the rural population was classified as unskilled, about double the percentage in the urban population. Urban–rural differences in the distribution of employment skill level were statistically significant (*p* < 0.001) and remained highly significant with adjustments for education (*p* = 0.006), gender (*p* < 0.001), SES (*p* < 0.001), alone and in combination (*p* < 0.001).

**Fig. 3:**
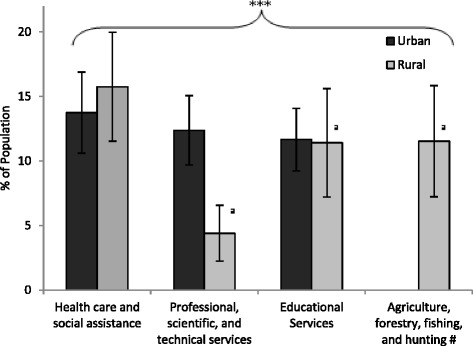
Occupational industry, based on NAICS classification, for urban and rural populations (≥18 yrs). Bars represent population weighted estimates ± 95 % CI. Difference between urban and rural is significant (***p < 0.001). ^a^High sampling variability, interpret with caution. # Data for Urban category is suppressed due to high sampling variability

NAICS industrial classification was also compared for employed respondents aged ≥18 yrs (Fig. 3). The three most common industries for urban employment were: health care and social assistance (13.8 %); professional, scientific, and technical services (12.4 %); and, educational services (11.7 %). Employment in health care and social assistance (15.8 %) and educational services (11.4 %) were also prevalent in the rural population. Employment in agriculture, forestry, fishing and hunting was the second most prevalent industrial group (11.5 %) in the rural population, while only a small portion (4.4 %) of the rural population was employed in professional, scientific, and technical services. Urban–rural differences in the distribution of employment industry were statistically significant (*p* < 0.001) and this difference was maintained with adjustment for each of education, gender, and SES alone, but not in combination (*p* = 0.985).

Analysis of total household income before tax revealed that 22.9 % of urban households (95 % CI: 20.0–25.8 %) and 18.9 % (95 % CI: 15.2–22.5 %) of rural households were considered low-income (household income at or below LICO) (*p* = 0.095).

### Potential chemical exposures

**Fig. 4:**
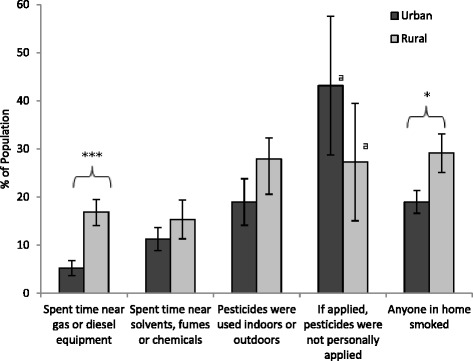
Prevalence of potential exposures to environmental pollutants among urban and rural populations. Bars represent population weighted estimates ± 95 % CI. Difference between urban and rural is significant (***p < 0.001). ^a^High sampling variability, interpret with caution

Other differences in urban and rural activities may also lead to differential exposure to various sources of environmental contaminants (Fig. 4 and Table 2). The rural population more frequently reported spending time near gas or diesel powered equipment, excluding vehicles, (16.9 %) compared to the urban population (5.2 %) (*p* < 0.001); this difference was maintained with adjustment for education, gender, age and SES. A difference was also noted for smoking in the household, with a greater prevalence of smoking in rural (29.1 %) than urban homes (19.0 %) (unadjusted and adjusted for SES *p* < 0.001). Neither group had a high prevalence of exposure to solvents, fumes, or strong smelling chemicals (11.3 % and 15.3 % for urban and rural, respectively; *p* > 0.05). Pesticide usage during the summer was more frequent in rural than urban populations (unadjusted *p* = 0.040). Additionally, more urban respondents (43.1 %) indicated they did not personally apply pesticides (i.e. were applied professionally) compared to rural respondents (27.3 %), although this difference was also not statistically significant.

### Housing characteristics

**Fig. 5:**
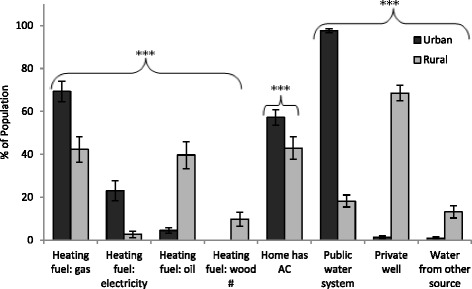
Household heating, cooling, and water source for urban and rural populations. Bars represent population weighted estimates ± 95 % CI. Difference between urban and rural is significant (***p < 0.001). # Data for Urban category is suppressed due to high sampling variability

The survey data revealed several differences in housing characteristics between urban and rural populations (Fig. 5 and Table 2). Specifically, differences in the distribution of home heating fuel were highly significant between the groups (unadjusted and adjusted for SES *p* < 0.001). The primary heating fuel in urban areas was gas (69.3 %), followed by electricity (23.1 %) and oil (4.5 %). In rural areas, gas (42.3 %) and oil (39.7 %) were the main fuels, with some residences using wood (9.8 %) and electricity (2.8 %). Additionally, air conditioning was more prevalent in urban (57.2 %) compared to rural homes (43.0 %) (unadjusted and adjusted for SES *p* < 0.001). The distribution of water sources also differed substantially between the groups (unadjusted and adjusted for SES *p* < 0.001). As anticipated, a large majority of the urban population (97.6 %) reported using a public water system for household water. In comparison, private wells (68.6 %) were the main water source in rural areas, with small contributions from public water systems (18.2 %) and use of other sources (e.g. bottled water; 13.2 %).

### Health status

Prevalence of self-reported asthma and heart disease (“Has your doctor told you that you have…”) was similar for urban and rural populations (Table 3). For all ages, the prevalence of asthma was similar between urban (11.1 %) and rural (10.4 %) groups. Among those 0–19 yrs, the prevalence of asthma was 12.0 % and 15.0 %, for urban and rural, respectively. In those 20+ yrs, the prevalence was slightly decreased at 10.8 % (urban) and 8.8 % (rural). Urban–rural differences were not significant for either age group. There was a slightly greater prevalence of chronic bronchitis/ emphysema in rural (4.9 %) compared to urban (3.2 %) areas which was significant only with adjustment for age (*p* = 0.024).

**Table 3:** Unadjusted and adjusted odds ratio (95 % confidence interval) estimates for the association between rural vs. urban residence and self-reported health status (reference group is urban)

Adjustment	Asthma	Heart disease	Chronic bronchitis or emphysema
None	0.9	1.2	1.5
	(0.7–1.3)	(0.8–1.7)	(0.9–2.5)
Education	0.8	1.0	1.0
	(0.5–1.1)	(0.7–1.5)	(0.6–1.6)
Gender	0.9	1.2	1.5
	(0.7–1.3)	(0.8–1.7)	(0.9–2.5)
Age	0.9	1.1	1.5
	(0.7–1.3)	(0.7–1.5)	(0.9–2.4)
SES	0.9	1.3	1.6
	(0.7–1.3)	(0.8–1.9)	(0.9–2.7)
Smoking	0.9	1.1	1.2
	(0.7–1.3)	(0.8–1.6)	(0.8–2.1)
All	0.8	1.1	1.0
	(0.5–1.2)	(0.7–1.7)	(0.6–1.8)

## Discussion

The main purpose of this study was to evaluate urban–rural differences in time-activity patterns, occupational activity, and housing characteristics using data collected in CHAPS 2. The survey data identified a number of differences that could contribute to the reported health disparity between urban and rural populations [1–6].

As previously reported [7], daily time-activity patterns indicated that rural populations spend significantly more time outdoors than urban populations. In the present analysis, the greatest differences were observed for adults and seniors. This likely reflects differences in occupation; over 10 % of employed people aged ≥18 yrs in rural areas work in primary industry (i.e. agriculture, forestry, fishing, and hunting) and rural respondents were almost twice as likely to report working outdoors compared to urban respondents. Being engaged in outdoor work is associated with increased exposure to ambient air pollution and also increased pollutant dose, as these activities are likely more vigorous requiring a greater inhalation rate [27]. Rural children spent more time outdoors which may reflect both greater outdoor play and chores. In comparison, a difference in daily time outdoors was not observed for adolescents (12–19 yrs), which may be attributable to similar amounts of free time spent as screen time (combined television, computer and video games) for urban and rural youth in Canada [28]. Although the differences in daily time-activity are small, incorporating time-activity patterns has been demonstrated to improve exposure estimates compared to estimates based solely on ambient air quality data [29, 30]. Sedentary behaviour is also an important risk factor for several chronic diseases. While we have not specifically quantified sedentary time, an analysis based on the Canadian Community Health Survey found little difference in self-reported leisure time physical activity between urban and rural residents [1].

In rural areas, ambient air quality is impacted by local activities including wood burning [31, 32]. It has been estimated that woodsmoke accounts for as little as 10 % and up to 70 % of provincial fine particulate matter (PM_2.5_) emissions in Canada [15]. In our analysis, wood was identified as the primary heating fuel for about 10 % of the rural residences compared to <0.1 % for urban areas. (NB: the calculated estimate for urban areas cannot be reported due to high sampling variability.) The burning of wood in stoves and fireplaces can produce significant quantities of PM_2.5_, carbon monoxide and nitrogen oxides. These pollutants can impact local air quality and are associated with health effects including adverse respiratory symptoms in children, lung function decrements, and increased emergency room visits [reviewed in 15]. Additionally, use of residential wood burning stoves and fireplaces increases indoor levels of PM_2.5_ directly from the appliance itself to the indoor environment. As well, vented PM_2.5_ can infiltrate from outdoors into the home [32].

Despite relatively low ambient levels of PM_2.5_ in rural areas, recent studies have associated PM_2.5_ with adverse health effects. 0.93 % of all-cause mortality among adults was attributed to anthropogenic PM_2.5_ (from forestry-related industry, wood burning stoves, and traffic) in rural British Columbia [31]. In a recent national Canadian study, in which 27 % of subjects lived in rural areas, significant associations of PM_2.5_ with non-accidental and cardiovascular mortality were reported [33]. PM_2.5_ was also associated with cardiovascular mortality in men residing in rural areas from the US Agricultural Health Cohort [34]. These studies demonstrate that ambient PM_2.5_ has a measurable effect on human health, even at the low level encountered in rural areas. A recent study also found the association between ozone and mortality was higher in rural areas than urban areas in the northeastern United States [35]. Indeed, it has also been suggested that the slope of the exposure-response relationship between air pollution and mortality may be steeper at lower concentrations [36]. This may be associated with increased exposure to ambient air pollution due to increased time spent outdoors potentially engaged in vigorous physical activity. Although the CHAPS 2 survey did not identify significant urban–rural differences in the prevalence of cardiac and respiratory diseases, there may be undetected differences in population vulnerability to air pollution. Other studies have demonstrated a greater incidence and prevalence of morbidity in rural populations [1–6].

Other environmental health factors may also contribute to the health disparity between urban and rural populations. For example, our data indicated that the rural population was significantly less likely to have air conditioning. Air conditioning use is associated with reduced household air exchange rates [37] and reduced infiltration of PM_2.5_ into residences [38]. Conversely, homes that rely on window opening for ventilation and cooling purposes have greater air exchange rates and increased infiltration of PM_2.5_. The presence and usage of air conditioning has been identified as a factor which reduces risks of adverse health impacts from air pollution [39–41]. It has also been identified as a factor in reduced risks of health effects and mortality associated with temperature and extreme heat events [42–44]. However, the potential for the presence and usage of air conditioning to modify health outcomes has not been evaluated in rural areas. Household smoking prevalence was also significantly greater among the rural (29.1 %) compared to the urban (19.0 %) populations in our study. The 2011 Canadian Tobacco Use Monitoring Survey indicated that smoking prevalence among Canadians ≥15 years was 17.3 % (95 % CI: 16.2–18.4 %) [45]. The household smoking rate of urban areas from CHAPS 2 is in agreement with this survey, and emphasizes the greater prevalence of this known health risk factor in rural areas [1–3]. Non-significant differences in pesticide usage were found in our analysis. This may be because CHAPS 2 did not differentiate between agricultural and household usage. Children living in agricultural communities have been reported to have greater tissue levels of biomarkers associated with pesticide exposure compared to urban counterparts [46].

Not surprisingly, we found that rural populations were more likely to be employed in agriculture, forestry, fishing and hunting. Also, more rural employment was categorized as unskilled. While it is well documented that employment in these occupations and industries is associated with greater risk of injury [16, 17], it is also more physically demanding, resulting in greater doses of inhaled pollutants, and as we observed, may involve greater exposure to gasoline and diesel powered equipment. However, there is evidence of a health benefit of physical activity, despite increased exposure to air pollution [47]. Overall, the differences reported in this manuscript could also be used to support health messaging targeted for rural populations, highlighting the potential for greater exposure and dose among rural populations in relation to greater time spent outdoors, higher prevalence of strenuous outdoor work, and reduced prevalence of air conditioning, particularly in rural areas prone to regional air pollution episodes.

24-h recall diaries, like the approach used in CHAPS 2, are the standard instrument for measuring time-activity patterns. Reproducibility of data has been established in previous studies [48, 49]. Use of additional objective measures such as global positioning system devices has been suggested to increase accuracy of location data [50], but this is not feasible in large studies and does not capture activity data.

Survey representativeness determines the generalizability of the results to the target populations. CHAPS 2 specifically targeted people living in five urban and two rural communities, and results may not be generalizable outside these areas. Previous studies have attributed urban–rural health disparities to differences in income and SES [1, 6]. However we found no significant difference in prevalence of low income between urban and rural areas. This is likely a reflection of our use of LICO values to evaluate household income, which increase with increasing community and household size. As a result, the LICO values for rural areas are thousands of dollars less than for the urban centres for the same household size. For industry of work classification, CHAPS 2 results corresponded well with data collected in the 2011 Canadian Census with a small overrepresentation (<5 %) of people in educational services. Despite efforts to ensure that rural respondents were truly rural, about 18 % of the rural population indicated that their household water source came from a public water system and about 40 % had natural gas as the main furnace fuel. These responses suggest that the rural sample included people living in areas close to larger communities, such that households had access to public water systems and natural gas. These individuals may resemble those living in urban areas with respect to many socioeconomic factors (e.g. income, education, occupation) which could have attenuated differences observed in our study.

## Conclusions

Analysis of data collected in CHAPS 2 revealed a number of differences in time-activity patterns, occupational activity, and housing characteristics between urban and rural populations. In particular, rural populations spent more time outdoors, were more likely to work outdoors and spent more time near gas or diesel powered equipment (other than vehicles). There was also a greater prevalence of wood burning as the main source of household heating, as well as a lower prevalence of air conditioning and a higher prevalence of smoking in rural areas. Additional research is warranted to better understand precisely how these differences may contribute to health disparities between urban and rural areas.

## Abbreviations

CATI: computer-assisted telephone interview
CHAPS: Canadian Human Activity Pattern Survey
CI: confidence interval
LICO: low income cut-offs
NAICS: North American Industrial Classification System
NOC-S: National Occupational Classification for Statistics
PM: particulate matter
PM_2.5_: fine particulate matter (<2.5 μm in diameter)
SES: socioeconomic status
US EPA: United States Environmental Protection Agency
yrs: years
